# Real-world comparison of lymphopenia profiles in S1P receptor modulators for multiple sclerosis: a multicenter retrospective study

**DOI:** 10.1007/s00415-025-13300-z

**Published:** 2025-08-06

**Authors:** Giorgia Teresa Maniscalco, Maddalena Sparaco, Maria Di Gregorio, Giuseppina Cafasso, Elisabetta Signoriello, Felice Romano, Rosa Iodice, Roberta Fantozzi, Paolo Bellantonio, Aurora Zanghi, Leonardo Sinisi, Alessandro D’Ambrosio, Vincenzo Busillo, Valentina Scarano, Rocco Capuano, Luigi Lavorgna, Michela Williams, Antonio De Martino, Maria Elena Di Battista, Daniele Di Giulio Cesare, Giacomo Lus, Emanuele Cassano, Paola Sofia Di Filippo, Grazia Sibilia, Gianmarco Abbadessa, Vincenzo Andreone, Simona Bonavita

**Affiliations:** 1 Neurological Clinic and Stroke Unit and Multiple Sclerosis Center “A. Cardarelli” Hospital, Naples, Italy; 2 Second Division of Neurology, Department of Advanced Medical and Surgical Sciences, University of Campania Vanvitelli, Naples, Italy; 3 Neurology Unit, University Hospital “San Giovanni Di Dio E Ruggi D’Aragona”, Salerno, Italy; 4 Department of Neurology, AORN San G. Moscati, Avellino, Italy; 5 Neurological and Stroke Unit–Multiple Sclerosis Center, CTO Hospital, AORN Ospedale Dei Colli, Naples, Italy; 6 Department of Neurosciences, Reproductive and Odontostomatological Sciences, Federico II University, Naples, Italy; 7 IRCCS Neuromed, Pozzilli, Isernia Italy; 8 Department of Medical and Surgical Sciences, University of Foggia, Foggia, Italy; 9 Department of Neurology and MS Center, San Paolo Hospital, Naples, Italy; 10 Department of Advanced Medical and Surgical Sciences, University of Campania “Luigi Vanvitelli”, Naples, Italy; 11 Ms Center, Maria SS. Addolorata Hospital, ASL Salerno, Eboli, Italy; 12 Department of Translational Medical Sciences, Section of Clinical Immunology, University of Naples “Federico II”, Naples, Italy; 13 Department of Neurology, “Ospedale del Mare”, Naples, Italy

**Keywords:** Multiple sclerosis, Sphingosine-1-phosphate receptor modulators, Lymphopenia

## Abstract

**Introduction:**

Sphingosine-1-phosphate (S1P) receptor modulators, regulating the S1P/S1PR pathway, lead to lymphocyte sequestration in lymphoid organs, which results in peripheral lymphopenia. This study evaluates the degree of lymphopenia induced by S1P modulators in people with Multiple Sclerosis (MS): Ozanimod, Siponimod, Ponesimod, and Fingolimod.

**Methods:**

We conducted a retrospective multicenter study across thirteen MS centers in Italy, including 191 MS patients (mean age 46.4 years; 61.3% women). Of these, 28.8% received Siponimod, 26.2% Ozanimod, 24.1% Fingolimod, and 20.9% Ponesimod. Lymphocyte counts were measured at baseline (T0), one month (T1), three months (T3), and six months (T6) post-treatment. Lymphopenia grades range from 0 (≥ 1.0 × 10^9 cells/L) to 4 (< 0.2 × 10^9 cells/L), according to Common Terminology Criteria for Adverse Events.

**Results:**

At T1, Ozanimod showed a significantly higher mean lymphocyte count than Siponimod and Fingolimod (p < 0.001), Ponesimod higher mean lymphocyte count than Siponimod (p = 0.006). The differences persisted between Ozanimod than Siponimod and Fingolimod at T3 (p = 0.01 and p = 0.04), and between Ponesimod and Siponimod at T6 (p = 0.03). Severe lymphopenia cases were significantly higher in Siponimod than in Ponesimod and Ozanimod, at T1 (p = 0.001 and p = 0.0001). The differences remained significant between Ozanimod and Siponimod, at T3 (p = 0.001), and between Ponesimod and Siponimod, Fingolimod and Ozanimod, at T6 (p = 0.001). Grade 4 lymphopenia was observed in Ozanimod and Siponimod at T3.

**Conclusion:**

This is the first real-world, head-to-head observational study comparing lymphopenia among different S1P modulators. Our results might assist in therapy choice depending on patients baseline hematological characteristics.

## Introduction

Sphingosine-1-phosphate (S1P) is a bioactive sphingolipid produced by sphingosine kinase, and it interacts with five S1P receptor subtypes (S1PR1-5) to regulate various cellular functions such as immune cell proliferation, survival, adhesion, and migration [1]. S1PR1, expressed on lymphocytes, controls the egress of these cells from lymphoid organs into the bloodstream, thereby playing a pivotal role in lymphocyte trafficking [2]. Due to this function, S1P receptor modulators, which sequester lymphocytes in lymphoid tissues, have been developed and employed in autoimmune diseases like multiple sclerosis (MS).

Several S1P modulators have been approved for MS treatment: Fingolimod (FTY720) [3, 4], Siponimod (BAF312) [5], Ozanimod (RPC1063) [6, 7], and Ponesimod (ACT-128800) [8]. These drugs are effective in reducing brain lesions, annualized relapse rate and slowing disease progression [3–8]. However, they vary in their receptor affinity profiles, with Fingolimod targeting S1PR1, S1PR3, S1PR4, and S1PR5 [9], while Siponimod and Ozanimod selectively engage S1PR1 and S1PR5 [10], and Ponesimod is a selective S1PR1 modulator [11]. This differential receptor targeting may influence the degree of lymphocyte sequestration and resultant lymphopenia, which is a known safety concern.

This study aims to compare the degrees of lymphopenia induced by these S1P modulators in a real-world cohort of MS patients.

## Methods

We conducted a retrospective, multicenter study across 13 MS centers in Italy. The inclusion criteria were based on a diagnosis of MS according to the McDonald criteria [12] and ongoing treatment with one of the four approved S1P modulators, as part of routine clinical care. Patients previously treated with another S1P modulator were excluded. Lymphocyte counts were measured locally, in keeping with the observational, real-world nature of the study., at baseline (T0), one month (T1), three months (T3), and six months (T6) following the initiation of S1P modulator therapy, based on clinical practice for monitoring Lymphopenia was defined and graded according to Common Terminology Criteria for Adverse Events (CTCAE) [13] as follows: grade 0 (normal): ≥ 1.0 × 10^9^ cells/L; grade 1: 0.8–0.999 × 10^9^ cells/L; grade 2: 0.5–0.799 × 10^9^ cells/L; grade 3: 0.2–0.499 × 10^9^ cells/L; grade 4: < 0.200 × 10^9^ cells/L. The study was submitted to the local ethics committee and conducted in accordance with the Declaration of Helsinki.

### Statistical analysis

Mean values and standard deviation (sd) were calculated for numerical variables such as age, Expanded Disability Status Scale (EDSS) score, disease duration and lymphocyte count.

Data were analyzed using descriptive statistics. Shapiro–Wilk test was used to assess the normality of continuous variables. The Chi-square test was used to examine differences in categorical variables. To analyze differences in mean lymphocyte values at different time points (T0, T1, T3, and T6), an Analysis of Covariance (ANCOVA) was conducted, adjusting for baseline covariates such as age, disease duration, EDSS, sex, and previous DMTs. Post hoc tests were performed to assess differences between the various S1P modulators treatment. Effect sizes were evaluated using Cohen’s d. Statistical significance was defined as p < 0.05. All analyses were conducted using Jamovi version 2.3.18.

## Results

### Cohort characteristics

According to inclusion and exclusion criteria, a total of 191 pwMS were enrolled in the study. Table 1 shows the demographic and clinical variables at baseline of our MS population. Mean (sd) age was 46.4 (± 11.8) years. Women were predominant (n = 117, 61.3%). The mean time (sd) since diagnosis was 127 (± 113) months.

**Table 1:** Patient demographics and clinical characteristics

Patients cohort	n = 191	Ozanimod (n = 50)	Siponimod (n = 55)	Ponesimod (n = 40)	Fingolimod (n = 46)	*p *value
% of total		26.2%	28.8%	20.9%	24.1%	> 0.05
*Sex*, *n* (%)						0.35
Female	117 (61.3)	26 (52)	33 (60)	27 (67.5)	31 (67.4)	
Male	74 (38.7)	24 (48)	22 (40)	13 (32.5)	15 (32.6)	
Age, mean (± sd)	46.4 (± 11.8)	46 (± 11.6)	54.8 (± 7.7)	44.4 (± 11.8)	38.5 (± 9.8)	** < 0.001**
Disease duration, mean (± sd) (months)	127 (± 113)	106 (104)	199 (111)	120 (113)	72 (77.6)	** < 0.001**
*MS type*, *n* (%)						** < 0.001**
Relapsing Remitting (RRMS)	142 (74.3)	48 (96)	10 (18.2)	40 (100)	44 (95.7)	
Primary Progressive (PPMS)	2 (1.1)	0	2 (3.6)	0	0	
Secondary progressive (SPMS)	47 (24.6)	2 (4)	43 (78.2)	0	2 (4.3)	
EDSS, median (25–75 IQR)	2.0 (3.0)	1.5 (1.5)	6 (1.88)	2 (1.0)	2 (1.0)	** < 0.001**
*Prior DMT*, *n* (%)						
Platform	74 (38.7)	12 (24)	15 (27.3)	13 (32.5)	34 (74)	
Naive	46 (24.1)	19 (38)	10 (18.2)	10 (25)	7 (15.2)	
DimethylFumarate	29 (15.2)	7 (14)	9 (16.4)	11 (27.5)	2 (4.3)	
Teriflunomide	21 (11.0)	7 (14)	9 (16.4)	5 (12.5)	0	
Natalizumab	9 (4.7)	2 (4)	4 (7.3)	0	3 (6.5)	
Ocrelizumab	5 (2.6)	1 (2)	4 (7.3)	0	0	
Other	4 (2.1)	0	3 (5.4)	1 (2.5)	0	
Cladribine	3 (1.6)	2 (4)	1 (1.7)	0	0	

The bold text indicates statistically significant values

142/191 patients (74.3%) were diagnosed with Relapsing Remitting MS (RRMS), 47 (24.6%) had Secondary Progressive MS (SPMS) and 2 (1.1%) Primary Progressive MS (PPMS). Median (IQR) EDSS was 2.0 (3.0).

55/191 (28.8%) received Siponimod, 50/191 (26.2%) Ozanimod, 46/191 (24.1%) Fingolimod and 10/191 (20.4.2%) Ponesimod.

Before S1P initiation, 74 (38.7%) patients were previously treated with platform therapies, 46 (24.1%) were naive, 29 (15.2%) had been treated with dimethylfumarate, 21 (11.0%) with teriflunomide, 9 (4.7%) with natalizumab, 5 (2.6%) with ocrelizumab, 3 (1.6%) were priorly treated with cladribine and 4 (2.1%) with other DMTs.

### Analysis of mean lymphocyte counts

**Fig. 1:**
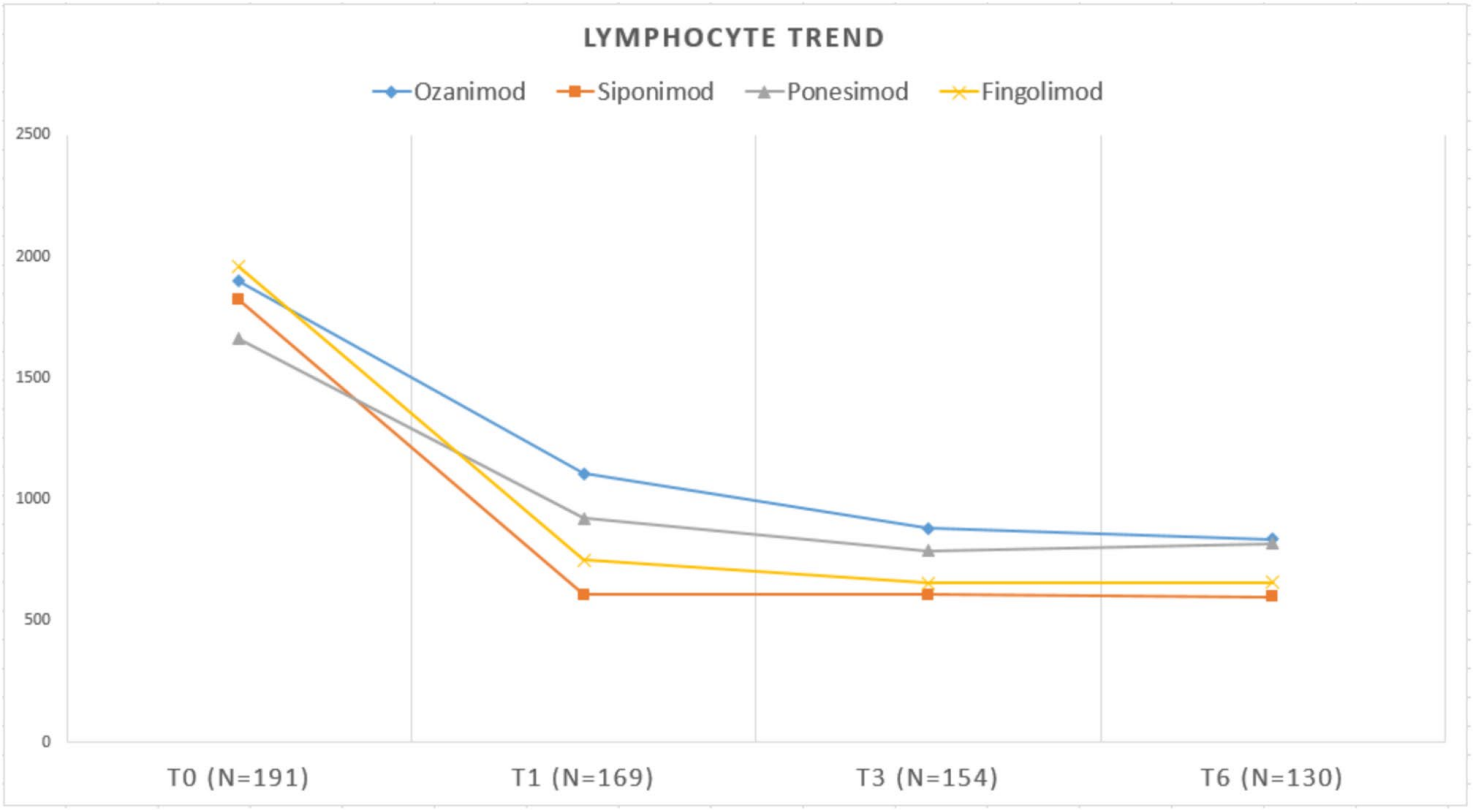
Trend of lymphocyte counts at the four timepoints

The trend of lymphocyte counts at the four timepoints is summarized in Fig. 1.

The differences between lymphocyte counts among the S1P modulators are shown in Table 2.

**Table 2:** ALC for different S1P modulators

S1P type	Baseline (T0) (n = 191)	T1 (n = 169)	T3 (n = 154)	T6 (n = 130)
Ozanimod, mean (± sd)	1899 (± 837)	1105 (± 521)	880 (± 431)	837 (± 476)
Siponimod, mean (± sd)	1822 (± 784)	608 (± 277)	607 (± 346)	598 (± 340)
Ponesimod, mean (± sd)	1662 (± 671)	921 (± 355)	790 (± 377)	818 (± 263)
Fingolimod, mean (± sd)	1958 (± 947)	751 (± 314)	656 (± 237)	659 (± 317)
*Post-hoc test (Cohen’s d)*				
Ozanimod—Siponimod	0.49	** < 0.001 (1.28)**	**0.01 (0.78)**	0.07 (0.66)
Ozanimod—Fingolimod	0.70	** < 0.001 (0.87)**	**0.04 (0.55)**	0.23 (0.35)
Ponesimod-Siponimod	0.94	**0.006 (0.89)**	0.07 (0.56)	**0.03 (0.79)**
Fingolimod-Siponimod	0.35	0.23 (0.40)	0.50 (0.22)	0.39 (0.31)
Ponesimod—Fingolimod	0.25	0.06 (0.48)	0.21 (0.33)	0.12 (0.48)
Ozanimod- Ponesimod	0.40	0.10 (0.38)	0.41 (0.21)	0.68 (0.13)

The bold text indicates statistically significant values

At baseline, there were no significant differences in mean lymphocyte counts among the four S1P modulators. As expected, all S1P modulators induced the greatest reduction in lymphocyte counts within the first month of treatment (T0 to T1). Throughout the observation period (T1 to T6), siponimod had the lowest lymphocyte values, while ozanimod had the highest.

At T1, ozanimod showed a significantly higher mean lymphocyte count than both siponimod (1105 vs. 608; p < 0.001) and fingolimod (1105 vs. 751; p < 0.001). Another difference was highlighted between ponesimod and siponimod (921 vs. 608; p = 0.006).

At T3 the different lymphocyte counts between ozanimod and siponimod were persistently significant (p = 0.01), and between ozanimod and fingolimod (p = 0.04).

At T6, ponesimod showed significantly higher lymphocyte count than siponimod (818 vs. 598; p = 0.03).

### Analysis of severe lymphopenia

**Fig. 2:**
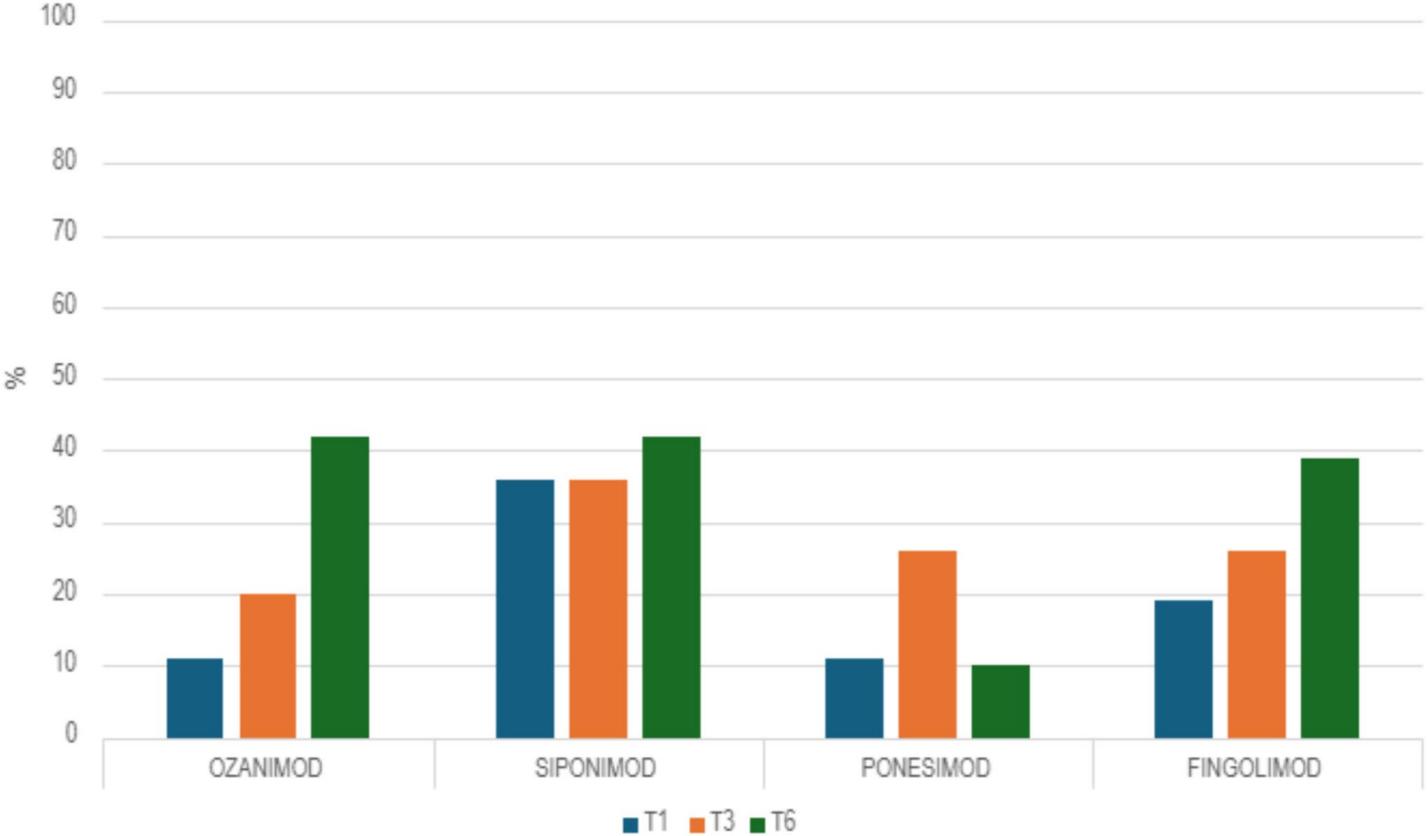
Combined percentage of cases presenting severe lymphopenia (Grade 3 + Grade 4) attributed to different S1P modulators at timepoints T1, T3, and T6

The study also assessed the incidence of severe lymphopenia (grades 3 and 4) among patients treated with the S1P modulators (Fig. 2 and Table 3).

**Table 3:** Comparison of severe lymphopenia (grade 3 + grade 4) among S1P modulators at T1, T3, T6

	T1 p value	T3 p value	T6 p value
Ozanimod- Fingolimod	0.28	0.56	0.79
Siponimod-Fingolimod	0.10	0.29	0.31
Ponesimod-Fingolimod	0.29	0.97	**0.01**
Ponesimod-Ozanimod	0.97	0.6	**0.01**
Ponesimod- Siponimod	**0.01**	0.3	**0.01**
Ozanimod- Siponimod	** < 0.01**	**0.01**	0.98

The bold text indicates statistically significant values

At T1, cases of severe lymphopenia were significantly higher in siponimod than in ponesimod and ozanimod (p = 0,001 and p = 0,0001). At T3, the difference remained significant only between ozanimod and siponimod (p = 0,001). At T6, ponesimod had significantly less cases of severe lymphopenia compared to siponimod, fingolimod and ozanimod (p = 0,001).

**Fig. 3:**
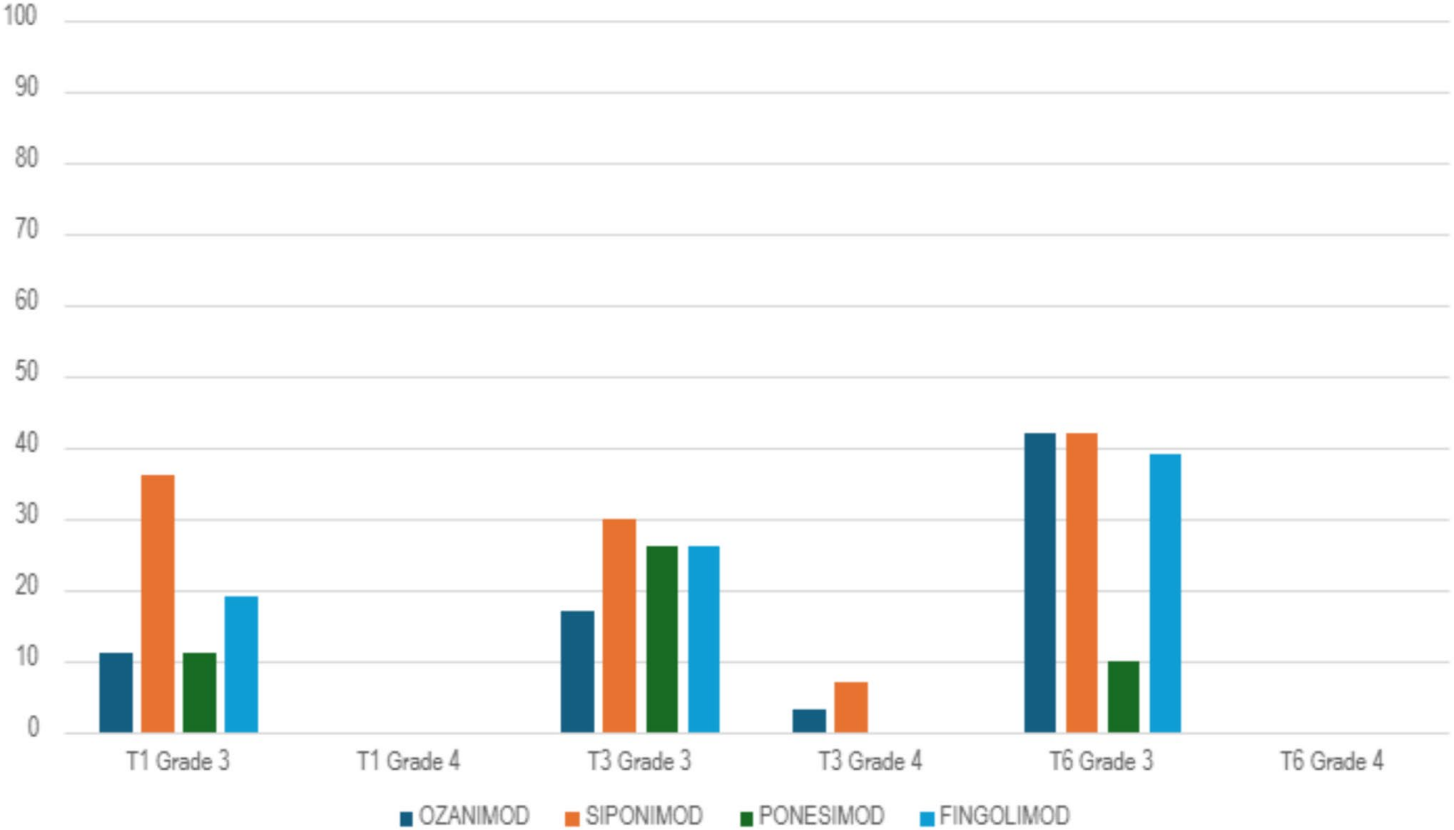
Percentage of cases with grade 3 and grade 4 lymphopenia separately reported across different S1P modulators at timepoints T1, T3, and T6

We also considered grade 3 and grade 4 separately (Fig. 3 and Table 4).

**Table 4:** Comparison of grade 3 and grade 4 lymphopenia among S1P modulators at T1, T3, T6

	T1 p value		T3 p value		T6 p value	
	Grade 3	Grade 4	Grade 3	Grade 4	Grade 3	Grade 4
Ozanimod- Fingolimod	0.28	-	0.37	-	0.79	-
Siponimod-Fingolimod	0.10	-		-	0.31	-
Ponesimod-Fingolimod	0.29	-	0.97	-	**0.01**	**-**
Ponesimod-Ozanimod	0.97	-		-	**0.01**	**-**
Ponesimod- Siponimod	**0.01**	**-**		-	**0.01**	**-**
Ozanimod- Siponimod	** < 0.01**	**-**		0.44	0.98	-

The bold text indicates statistically significant values

All S1P modulators induced grade 3 lymphopenia, at each time point (from T1 to T6), with cases of grade 3 lymphopenia significantly higher in siponimod than in ponesimod and ozanimod (p = 0,001 and 0,0001) at T1.

At T6, ponesimod had significantly less cases of grade 3 lymphopenia compared to siponimod, fingolimod and ozanimod (p = 0,001).

Grade 4 lymphopenia was induced only by ozanimod and siponimod at T3, and particularly in siponimod more than in ozanimod, although the difference is not statistically significant (p = 0.44)**.**

## Discussion

Our retrospective, multicenter study is the first real-world investigation to directly compare absolute lymphocyte counts (ALCs) across the four S1PR modulators approved for MS treatment. In a cohort of 191 MS patients, we analyzed mean ALCs over six months following treatment initiation and assessed the degree of lymphopenia associated with each S1P modulator.

S1P modulators exert their pharmacodynamic effects by sequestering lymphocytes within lymphoid tissues, via interaction with S1P receptors. However, the extent of lymphocyte reduction varies among the different agents, largely due to differences in receptor binding affinity. Fingolimod binds S1PR 1,3,4 and 5; siponimod and ozanimod selectively target S1PR 1 and S1PR 5. Ponesimod, the most selective agent, exclusively binds S1PR 1.

Although lymphocyte reduction is not directly with therapeutic efficacy [14, 15], it raises safety concerns, particularly regarding increased susceptibility to viral infections [16]. Fingolimod trials have demonstrated that ALC nadirs are influenced by baseline lymphocyte levels, sex, and BMI [17]. Regulatory trials reported mean nadirs of approximately 500/μL for fingolimod, 800/μL for ozanimod, 560/μL for siponimod, and 650/μL for ponesimod [18]. The corresponding relative lymphocyte reductions were 70–80% for fingolimod and siponimod, 55% for ozanimod, and 60–70% for ponesimod [18]. Our study is the first to directly compare all four S1P modulators in a real-world context, as no head-to-head clinical trials have been conducted. All agents induced the greatest reduction in ALCs within the first month of treatment and levels remained below the normal range throughout the six-month observation period. At six months mean ALC reductions were 66.3% for fingolimod, 55.9% for ozanimod, 67.2% for siponimod, 50.8% for ponesimod, findings consistent with previous studies.

Siponimod was associated with significantly lower mean ALC compared to both ozanimod, (at months 1 and 3), and ponesimod, (at months 1 and 6).

Conversely, differences with fingolimod emerged earlier with ozanimod (at month 3). These results are in line with other recent findings [19].

Ozanimod was associated with a lower reporting frequency of lymphopenia compared to both siponimod and fingolimod. These results also align with previous findings. A comparative analysis of 1-year outcomes using matching-adjusted indirect comparisons, found ozanimod to be associated with significantly higher mean ALCs compared to fingolimod [20]. In Swallow et al. study, the difference between the two drugs in ALC was 0.4 × 10^9^/l, in our study at T1 it was 0.35 × 10^9^/l.

Grade 4 lymphopenia was reported in 1% of patients in both the EXPAND trial for siponimod [5] and the TRANSFORMS trial for fingolimod [4]. In the FREEDOMS trial, the incidence was 0.5% for for fingolimod [3]; for ozanimod, severe lymphopenia was observed in 2.5% and 4.2% of patients in the SUNBEAM and RADIANCE trials, respectively [6, 7]. The OPTIMUM trial for ponesimod [8] did not specify rates of grade 4 lymphopenia, although it was listed among the reasons for treatment discontinuation.

While clinical trial data indicate a range of 0.5–4.2% for severe lymphopenia, real-world studies suggest higher rates. For fingolimod, grade 4 lymphopenia has been observed in 2.2–15% of patients, and has been associated with low baseline ALC and prior treatment history [15, 21, 22]. Real-world studies on siponimod reported grade 4 lymphopenia in 8.5% of patients within the firt month of treatment, without significant clinical correlates [23]. Data on ozanimod and ponesimod in real world setting remain limited, although some insights have been drawn from studies on treatment transitions among S1P modulators [24, 25].

In our cohort, grade 4 lymphopenia occurred only in patients treated with ozanimod (2.9%) and siponimod (6.8%), consistent with findings from the SUNBEAM and RADIANCE trials and Gilmartin study [6, 7, 23]. In contrast, no cases of grade 4 lymphopenia were observed in patients treated with fingolimod, in line with the relatively low rates reported in pivotal trials [3, 4].

The differential severity and trajectory of lymphopenia associated with each S1PR modulator may guide clinicians in choosing appropriate therapy and tailoring patient monitoring strategies. Although hematologic monitoring guidelines remain consistent across all S1P modulators, the significantly lower lymphocyte counts and higher incidence of severe lymphopenia in patients treated with siponimod, especially early in treatment, compared to ponesimod and ozanimod, highlight the need for greater attention in hematologic monitoring in this subgroup. Identifying patients who are likely to develop Grade 4 lymphopenia, particularly with Ozanimod and Siponimod at T3, allows clinicians to proactively manage treatment plans based on individual risk.

### Limitations

Unlike randomized clinical trials, real-world studies reflect routine clinical practice and therefore encompass a broader spectrum of patient characteristics, including variability in age, disease duration, EDSS scores, and MS phenotypes. Notably, patients treated with siponimod tend to have a higher mean age, longer disease duration, and greater disability, consistent with its indication for SPMS. Nevertheless, in our study, there were no significant differences in baseline mean ALCs among the four treatment groups.

## Conclusions

This real-world analysis demonstrates significant variability in lymphocyte reduction across the four approved S1P receptor modulators. These findings offer valuable guidance for clinicians in tailoring S1P modulator therapy to individual patients, balancing efficacy with the risk of lymphopenia and potential infection. Further real-world studies are needed to evaluate the incidence and severity of long-term lymphopenia, and to explore its clinical implications, particularly infection risk and post-treatment lymphocyte recovery in pwMS receiving S1P modulators.
